# A State Board of Health

**Published:** 1900-04

**Authors:** 


### A State Board of Health.

Elsewhere in this issue we present the draft of a bill to create a State Board of Health in Texas, drawn by Chairman Dr. R. H. Harrison last year after it had become evident that the Twenty-sixth Legislature would take no action on that submitted by this committee (acting for the State Medical Association). The bill is crude, and needs a little touching up; for instance, no provision is made for any salary of any officer or member except the president, and it calls for no appropriation to carry it into effect. It is incomplete, in our opinion, in that it creates no inspectors in the several counties to co-operate with the State Board in the matter of enforcing local sanitation, such as the removal of sinks, surface privies, etc., cleansing of privy boxes, drainage, the abolition of surface wells or seepage water for drinking purposes, protection of streams from pollution; the disinfection of infected rooms, persons, and things, etc. But it furnishes an excellent basis upon which the State Association at the approaching session can construct a bill.

It will be observed that the bill recognizes the existing quarantine department as a feature of a State Board, and makes the chief quarantine officer (now called, improperly, "State Health Officer") ex-officio president of the Board, and leaves him alone in the management of quarantine, with discretion as to modifications where any relaxation can be safely made. No other member of the board has any say-so about quarantine. The Secretary of the Quarantine Department is to be the head of the Bureau of Vital Statistics, and there is to be a chemist and bacteriologist, and a veterinary surgeon or physician.

The general plan is precisely what the Journal has advocated for years; not because it is what we would rather have, but because it will do very well, and will be an improvement on the plan in operation, which is nothing but quarantine, and does not comprehend any of the other essentials of sanitation; and because we are much more likely to secure the passage of an act which recognizes the present status, and enlarges upon the existing system, adding other essentials, than we are to get even a hearing of any bill which proposes to abolish quarantine. The people of Texas are wedded to quarantine, and it is impregnable; it is entrenched behind a record of years of exclusion of foreign pestilence, and that is all they see.

Members of the Legislature should be made to distinctly understand that in asking for the passage of a bill for the further protection of the public health, the State Association is by no means making war upon the quarantine department, but aiming to strengthen the existing system by extending the scope of State supervision over local diseases, and seeking to remove and prevent, by the intelligent application of the principles of preventive medicine, the causes and extension of typhoid fever, diphtheria, dysentery, erysipelas, consumption, and other fatal maladies which carry off more victims than do yellow fever and cholera combined.

As a necessary preliminary to the successful application of State sanitation, of course the vital and mortuary statistics of the State must be collected, preserved, and studied. We have recently been informed that the Governor is much impressed with the importance of the last mentioned reform, and some hope exists that he may in his next message recommend some legislation along the lines indicated.

---

THE THIRTY-SECOND ANNUAL MEETING of the Texas State Medical Association will be held at Waco beginning Tuesday, April 24th inst. See call or announcement elsewhere by Dr. Gardner, the President. The season of the year—the most delightful—is propitious; Waco is central and easily accessible from everywhere; reduced rates have been secured on all lines and at all hotels; the people of Waco are delightfully hospitable; there is much important work to be done, and a most attractive program has been prepared, both for the meetings and socially. It is expected that every physician who feels that pride and interest in the profession which he ought to feel will make it his especial business to attend this meeting. Those who contemplate becoming members are advised to take with them their diplomas, to avoid trouble and delay. The Association is booming, prospering as in former days, and we hope soon to see every reputable physician in Texas enrolled.

---

DR. P. C. COLEMAN, of Colorado City, Mitchell county, Texas, ex-President Texas State Medical Association, is a candidate for Congress from his district. We hope he will be elected. He would make a good Congressman or a good anything else where intelligence, knowledge, integrity, and ability are required. He has a comprehensive knowledge of sanitation and would doubtless be of great service to this State in aiding the profession to secure sanitary legislation.

